# Gratitude Questionnaire–20 Items (G20): A Cross-Cultural, Psychometric and Crowdsourcing Analysis

**DOI:** 10.3389/fpsyg.2020.626330

**Published:** 2020-12-21

**Authors:** Gloria Bernabe-Valero, José S. Blasco-Magraner, Marianela R. García-March

**Affiliations:** ^1^Research Team Mind, Emotion, and Behavior Lab, Catholic University of Valencia San Vicente Mártir, Valencia, Spain; ^2^Colaborator Research Team Mind, Emotion, and Behavior Lab, University of Valencia, Valencia, Spain

**Keywords:** crowdsourcing platform, gratitude questionnaire, psychometry, positive psychology, reliability, validity, cross-cultural, English adaptation

## Abstract

The use in psychology of crowdsourcing platforms as a method of data collection has been increasing in popularity because of its relative ease and versatility. Our goal is to adapt the Gratitude Questionnaire–20 Items (G20) to the English language by using data collected through a crowdsourcing platform. The G20 is a comprehensive instrument that takes in consideration the different basic processes of gratitude and assesses the construct’s cognitive, evaluative, emotional, and behavioral processes. We test the psychometric properties of the English version of the G20 with a Prolific (ProA) user sample. We assess the adequacy of the G20 for the crowdsourcing population in its English version. A description of the characteristics of the participants is conducted. Reliability analyses reveal an optimal internal consistency of the adapted scale. The results are discussed from a cross-cultural vision of gratitude. We conclude that the Gratitude Questionnaire–20 Items (G20), adapted to English with an American sample, is a psychometrically strong instrument to measure gratitude using crowdsourcing platforms for data collection and, therefore, a reference and useful tool in future research.

## Introduction

Digitalization has revolutionized many areas of human life; specifically, research has advanced by leaps and bounds with all the new possibilities offered by the rapid changes in technology (Moret-Tatay et al., 2019). One advancement that has radically changed in the field of psychology has been the increased possibility of recruiting participants through different social networks and electronic devices (Harteis et al., 2020). These new possibilities of digitization have extended the variety of participant sample pools. A few decades ago, most of the participant samples came from college students (Hauser and Schwarz, 2016). Given that access to the student population was the most accessible for university researchers, the use of samples of university students represented important advances that allowed for the validation of numerous assessment instruments for the standard population. Despite their validity, these studies have not been exempt from criticism regarding possible biases due to certain characteristics of this population. Specifically, one of the handicaps with this type of sample was the age range, which in most cases ranged between 18 and 30 years. In contrast, today, the breadth of possible contacts is vast due to the great proliferation of social networks. At present, anyone can respond to a survey from their smartphone, tablet, or computer almost anywhere and at almost any time. Thus, crowdsourcing platforms have emerged that allow recruitment niches or representative samples on demand, becoming flexible tools for online research (Bakici, 2020). Furthermore, obtaining representative samples for studies in psychology becomes a more efficient process and can offer us advances in cross-cultural studies (Cuccolo et al., 2020). Therefore, the expansion of crowdsourcing platforms for use in psychological research opens a way to consolidate other options in addition to the university student population. It will be necessary to analyze the characteristics of the crowdsourcing samples and check how they behave psychometrically compared to student samples.

Crowdsourcing, as described by Majima et al. (2017), is an online activity in which users voluntarily take part in the collection of data by undertaking tasks proposed by the crowdsourcer and receive some kind of compensation, mostly monetary. These platforms are considered by Majima et al. (2017) quite useful for behavioral research because they offer the opportunity of collecting higher amounts of data with a higher diversity of participants and a faster theory and/or experiment cycle at a relatively low cost.

Several studies in recent years have explored the differences between the standard collection approach and crowdsourcing data collecting, concentrating mostly on Amazon MTurk as the main option. Most of these studies conclude that the positive effect in data collection is so significant, that it is advisable to use crowdsourcing for research studies; although more research is needed in order to fully understand the functioning of crowdsourcing in each of the different research themes, and also, in order to be able to control variables like: age, gender, naivety, personality traits, among others (Paolacci and Chandler, 2014; Choi and Lee, 2016; Majima et al., 2017; Peer et al., 2017; Probst et al., 2017; Stewart et al., 2017; Uhlmann et al., 2019).

Even though MTurk is considered as one of the most popular crowdsourcing platforms for research (Litman et al., 2017), some flaws have also been found. Goodman et al. (2013) found that MTurk users show lower rates of self-esteem and lower cognitive capacities. They seem to be less extraverted and emotionally less stable, and they lean toward higher materialism and value money more than time.

Regarding specific variables such as attention, reliability, naivety, demographics, or dishonest behavior and cheating, different studies compare MTurk with other crowdsourcing platforms like Prolific (ProA) or CrowdFlower (CF) and found that these alternative platforms offer viable possibilities. One important variable is demographics. Litman et al. (2017) found that ProA participants reside mainly in Europe and Asia, MTurk participants are mostly from the US, and participants recruited through CF are more ethnically diverse. Therefore, they advise researchers to consider the demographic variables when choosing which platform they will use since it will have an important effect on the population they reach.

Peer et al. (2017) consider lack of naivety to be an important problem with MTurk participants and find that ProA and CF offer the possibility of recruiting participants who present higher levels of naivety. These authors also find that participants recruited through ProA show higher levels of internal reliability than CF participants because of the latter’s higher failure rates in attention tasks. Participants through ProA show only slightly lower levels of attention than MTurk, which does not significantly affect the measurements of reliability. Also, it is observed that ProA participants are less prone to engage in dishonest behavior or cheating than participants from MTurk. In general, ProA participants show both a low dropout and a fast response rate. With a good attention level, the reliability rate is high, and the reproducibility is good. Even though the levels of dishonesty are medium, they are still lower than those found in MTurk. With all this, Peer et al. conclude that ProA is the best alternative to MTurk. Accordingly, for the present work, when analyzing the psychometric properties of the G-20 with participants recruited though a crowdsourcing platform, we have chosen ProA for data collection.

Our aim is to analyze the psychometric properties of the Gratitude Questionnaire–20 Items (G20) with participants recruited though ProA. Since Robert Emmons and Michael McCullough published their monographic work (Emmons and McCullough, 2004), The Psychology of Gratitude (2004) an expanding area has emerged that has made evident the importance of studying this construct evident. Various measuring instruments have been created and validated on different samples. The most widespread of all is the Gratitude Questionnaire—Six Items Form (GQ-6; McCullough et al., 2002). The GQ-6 is a six item self-report scale that focuses on the emotional component of the gratitude (Hudecek et al., 2020) and assesses individual differences in the tendency to experience gratitude in daily life. The GQ-6 has already been validated in several other languages including Hungarian (Tamás et al., 2014), Dutch (Jans-Beken et al., 2015), Chinese (Chen et al., 2008), Portuguese (Gouveia et al., 2019), Spanish (Bernabé-Valero et al., 2013), and German (Hudecek et al., 2020).

Another well-known instrument is the Gratitude Resentment and Appreciation Test (GRAT) (Watkins et al., 2003), a 16-item measure of dispositional gratitude. In recent years, more comprehensive instruments on gratitude have been created; i.e., the Multi-Component Gratitude Measure (MCGM) (Morgan et al., 2017) which also assesses attitudinal and behavioral aspects, and the Gratitude Questionnaire–20 item (G20, Bernabé-Valero et al., 2014) which is based on a conceptual and comprehensive delimitation of the concept of gratitude, being the latter questionnaire the object of analysis in the present study.

The construction of the G20 follows the proposed definition: “gratitude could be understood as a disposition to recognize, value, and respond to the positive aspects of personal existence, experienced as gifts received” (Bernabé-Valero et al., 2014, p. 279). This instrument has several benefits: the inclusion of various types of gratitude depending on the agent that arouses it, the affective valence of the object for which gratitude is elicited, and the inclusion of various manifestations (cognitive, emotional, and action tendencies).

As stated above, a wider definition of gratitude based on the psychological processes that lead to gratitude, was taken into account when developing the G20. This definition includes (1) the recognition of gifts, (2) the attribution to an agent, (3) the valuation of the object of gratitude, and (4) the manifestation of gratitude. In this regard, Emmons and McNamara (2006) emphasized the importance of studying the process leading to gratitude and supported the proposal that the four-step gratitude process was handled by limbic-frontal interactions (Damasio and Anderson, 2005). From this neurological point of view, it was proposed that for the recipient of a benefit, the four-step process would imply (1) recognizing that a gift has been received, (2) calculating benefits/costs associated with the gift, (3) experiencing an emotion that begins in appreciation and emerges into gratitude, (4) with memory of the benefit and benefactor as well as the emotion of gratitude initiating and sustaining a motivational state to reciprocate the benefit received (Emmons and McNamara, 2006, p 22.). Thus, neurological data supports that direct and indirect reciprocity of gratitude favor human cooperation by facilitating the return of a gift to a benefactor (Emmons and McNamara, 2006).

In relation to the agents toward whom gratitude is experienced, the G20 includes in its Interpersonal Gratitude (IG) scale expressions of gratitude toward other people with different degrees of closeness in the relationship (for example, family members and people who barely know each other), which provides a measure of gratitude focused on others. Transcendental Gratitude is measured in reference to the gratitude experienced toward transcendental forces (the existential referent of each person, such as God, luck, destiny, life, etc.). This type of Gratitude does not make explicit reference to any religion but encompasses all transcendent experiences from the most concrete and formalized to the most subjective.

The affective valence that the object of gratitude arouses is formulated by considering both the gratitude elicited through pleasant experiences, as well as the gratitude elicited based on the value a person assigns to experiences that generate suffering. These emotional aspects are measured through various scales, Recognition of Gifts (RG) for positive affective valence and Gratitude in the face of Suffering (GS) for negative valence.

Behavioral manifestations of gratitude are expressly evaluated through the Expression of Gratitude scale (EG). Other cognitive, emotional, and behavioral manifestations are explored throughout all the G20 scales.

Thus, the G20 is the focus of our interest due to the solid conceptual foundation on which it is based, as well as its excellent psychometric properties. This questionnaire, which was constructed for the Spanish population (Bernabé-Valero et al., 2014), has been adapted for the Argentine population (Klos et al., 2020) and translated into Portuguese with a Brazilian sample (Ribeiro-Viana, 2016).

Our purpose is to analyze the psychometric properties of the G20 questionnaire in English and assess the suitability of its use by users of crowdsourcing platforms.

## Materials and Methods

Three hundred and six (306) participants were recruited from the prolific platform ProA, with the condition that the entire sample be residents of the United States, whose main language is English. Four participants were excluded because they did not meet this criteria, leaving a sample of 302 participants of which 153 (51%) were women and 149 (49%) were men.

A cross-sectional design was used in which ages ranging from 19 to 82 years (*M* = 45.07, ST = 15.94) were represented. The participants by generational breakdown were: 22% between 18 and 29 years old, 17% between 30 and 39 years old, 15% between 40 and 49 years old, 21% between 50 and 59 years old and 25% were 60+ years and older. Regarding ethnicity: 8% were Asian; 15% Black; 5% were of mixed race, 3% other, and 69% white. Table 1 shows sociodemographic data such as: employment status, educational level, and marital status.

**TABLE 1:** Sociodemographic characteristics.

**Employment status**	**Percent**	**Educational level**	**Percent**	**Marital status**	**Percent**
A homemaker	4.30	Bachelor’s degree	42.38	Separated	4.30
A student	7.67	Doctorate degree	2.32	Single, never married	11.92
Other	1.99	Master’s degree	14.24	Widowed	13.91
Out of work and looking for work	12.58	No schooling completed	16.56	Married or domestic partnership	47.02
Out of work but not currently looking for work	3.31	Professional degree	2.65	Divorced	12.25
Retired	14.90	Trade/technical/vocational training	21.85		
Salaried	34.11				
Self-employed	17.22				
Unable to work	3.97				

To control the variables of *frequency of use* and *experience with* crowdsourcing platforms, one item was chosen, (Q1) Frequency of use of crowdsourcing platforms—How many hours do you dedicate to crowdsourcing platforms? (e.g., Prolific, MTurk, CrowdFlower) Indicate the approximate number of hours per week. Participants’ responses to this question ranged between 0.5 and 60 h per week (*M* = 8.55, *SD* = 9.29). Following these results, the group was divided into two categories, (a) low crowdsourcing, those participants who use these platforms 5 or less hours a week (*N* = 175), and (b) high crowdsourcing, those who use the platforms more than 5 h a week (*N* = 127).

### Instruments

For the adaptation of the G20 (Bernabé-Valero et al., 2014), a back-translation procedure was performed as recommended by the International Test Commission (ITC) and the previous literature (Muñiz et al., 2013). All original items were initially translated from Spanish to English by a native English speaker with a fluent command of Spanish, and then translated back into Spanish by another bilingual professional. The result was discussed among a group of four professionals with cross-cultural experience: two Americans, an Irish psychology professor and one of the authors, a Spanish psychologist with a high degree of international experience. For the few items that were identified as potentially mistranslated or unclear, an alternative translation was made to preserve the meaning of the item. Participants have to rate twenty items (e.g., “I feel grateful when someone I hardly know helps me and/or is kind to me”) on a seven-point Likert scale (1 = strongly disagree to 7 = strongly agree). The final version of the instrument appears in Supplementary Appendix A.

The G20 scale has 4 factors: (1) Interpersonal Gratitude (IG)—gratitude that is experienced toward other people when receiving a gif or an act of kindness. It refers to benefactors with different types of relationships to the beneficiary and focuses on the evaluative, emotional and behavioral elements of gratitude; (2) Gratitude in the face of Suffering (GS)—this factor refers to the integration of suffering in the concept of gratitude. It assesses the ability to understand situations of suffering as beneficial and to feel gratitude despite it. Likewise, it assesses if the person is able to move forward despite difficulties and to use gratitude as a resource for resiliency. It includes the cognitive-evaluative and emotional elements of gratitude; (3) Recognition of Gifts (RG)—awareness of the positive aspects of existence while considering them as gifts and implicitly attributing these gifts to a transpersonal agent (e.g., destiny, luck, nature, or divine providence). It includes the process that leads to the recognition of assets and their appraisement, as well as the social comparison that gives rise to the awareness of the positive aspects in one’s life; (4) Expression of Gratitude (EG): the experience and expression of gratitude toward transpersonal forces. Forms of expression can be verbal expression, rituals, and an attitude toward life of trying to be happy.

The G20 obtained good reliability indices in its construction with a Spanish sample. Cronbach’s alpha for each subscale was optimal (GI α = 0.84, GS α = 0.78, RD α = 0.75, EG α = 0.75).

To measure the convergent validity, Gratitude Questionnaire—Six Items Form (GQ-6; McCullough et al., 2002) was used. This questionnaire focuses on the emotional component of the gratitude (Hudecek et al., 2020) based on an understanding of the concept of gratitude as “a generalized tendency to recognize and respond with grateful emotion to the roles of other people’s benevolence in the positive experiences and outcomes that one obtains” (McCullough et al., 2002, p. 112). Item 6 was removed for theoretical and empirical reasons (see more in Chen et al., 2008; Bernabe-Valero, 2012; Hudecek et al., 2020). The final GQ-5 internal consistency was α = 0.89. Responses range from 1 to 7 on a seven-point Likert scale (1 = strongly disagree and 7 = strongly agree). Possible scores ranged from 5 to 35, with higher scores indicating a higher level of gratitude.

### Statistical Analysis

The analyses were developed through the SPSS 22 and Amos 18.0 module. Cronbach Alpha was employed to test the internal consistency and a confirmatory factor analysis (CFA) was carried out accompanied by the goodness of fit indices. No rotation of the data was employed. Confirmation of the adequacy of the model has been used within the absolute fit indices; the chi-square statistic X^2^, and its ratio among degrees of freedom where values under 2 are recommendable. In terms of incremental fit indices, the comparative fit index (CFI) was selected. This follows a range of values between 0 and 1 and the reference value is 0.90. Within parsimony adjustment indices, the error of the root mean square approximation (RMSEA) of the RMSR similarly, the smaller its value, the better the fit, the reference value being 0.05. Finally, an analysis of invariance was carried out.

## Results

The internal consistency of the scales obtained using Cronbach’s alpha, following the criteria of George and Mallery (2003), was excellent for GS (α = 0.92), good for IG (α = 0.88) and RG (α = 0.87), and acceptable for EG (α = 0.79).

Table 2 depicts the Mean, Standard Deviation, Skewness, and Kurtosis for each subfactor regarding the whole dataset, and each group under study.

**TABLE 2:** Means, standard deviation (SD), skewness, and kurtosis.

		**IG**	**GS**	**RG**	**EG**
Total	Mean	6.11	5.24	5.88	4.28
	SD	0.71	1.39	1.10	1.49
	Skewness	–1.7	–0.80	–1.31	–0.26
	Kurtosis	3.45	0.05	1.90	–0.93
Group 1	Mean	6.04	5.20	5.84	4.13
	SD	0.71	1.35	1.06	1.50
	Skewness	–1.28	–0.79	–1.06	–0.26
	Kurtosis	1.55	0.17	1.00	–0.84
Group 2	Mean	6.22	5.30	5.95	4.50
	SD	1.45	1.15	1.47	1.45
	Skewness	–2.45	–0.83	–1.64	–0.26
	Kurtosis	7.54	–0.04	3.07	–1.15

After a student test for independent samples, statistical significant differences were found for two subscales of Gratitude across groups: IG [*t*_(__300)_ = 2.27; *p* < 0.01; *d’* = 0.26] EG [*t*_(__300)_ = 2.14; *p* < 0.01; *d’* = 0.25].

Table 3 shows the descriptive statistics of the G-20 scale. Women achieved a higher mean score on the total G-20 scale and on the subscales IG, RG, and EG, and men scored higher on GS. The differences were significant in IG, *t*(300) = −3.309, *p* < 0.01.

**TABLE 3:** Mean, standard deviation (SD) of Gratitude by gender.

	**Total GQ20**		**IG**		**GS**		**RG**		**EG**	
	**Men**	**Women**	**Men**	**Women**	**Men**	**Women**	**Men**	**Women**	**Men**	**Women**
Mean	107.79	111.56	5.98	6.25	5.26	5.22	5.78	5.98	4.12	4.44
SD	18.45	17.69	0.77	0.62	1.34	1.45	1.13	1.06	1.53	1.45
Minimum	45.00	49.00	2.86	3.43	1.00	1.00	1.25	2.00	1.00	1.00
Maximum	136.00	136.00	6.71	6.71	7.00	7.00	7.00	7.00	6.50	6.50

To test the criterion validity, the G-20 was correlated with GQ-5. This was done through a zero-order correlation (see Table 4) and a partial correlation between the groups, as can be seen in Table 5. G-20 subfactors depicted divergent values for GQ-5. The comparison between zero and partial correlations seems to support similar results without or controlling the different groups. Of note, a lack of correlation was found between GQ-5 and the other subscales of G-20.

**TABLE 4:** Zero order Pearson correlation for the whole data set.

	**IG**	**GS**	**RG**	**EG**	**GQ5**
IG	–				
GS	0.47***	–			
RG	0.58***	0.75***	–		
EG	0.35***	0.54***	0.59***	–	
GQ5	0.03	0.07	0.03	0.05	–

*^∗^p < 0.05, ^∗∗^p < 0.01, ^∗∗∗^p < 0.001.*

**TABLE 5:** Partial Pearson correlation in the variable groups.

	**IG**	**GS**	**RG**	**EG**	**GQ5**
IG	–				
GS	0.47***	–			
RG	0.58***	0.75***	–		
EG	0.34***	0.54***	0.59***	–	
GQ5	0.012	0.06	0.03	0.03	–

*^∗^p < 0.05, ^∗∗^p < 0.01, ^∗∗∗^p < 0.001.*

**FIGURE 1:**
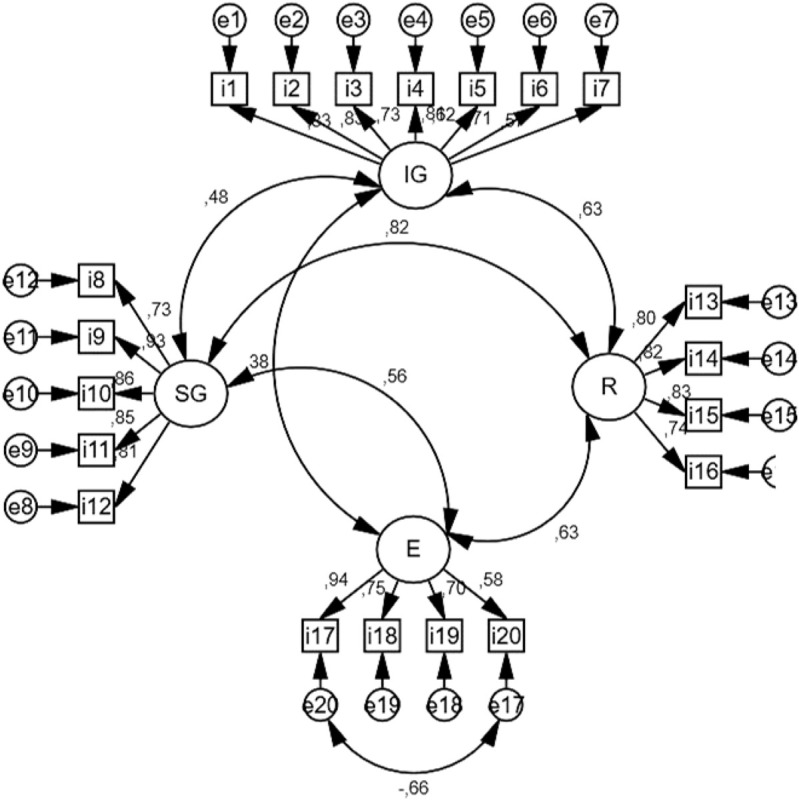
Factor loading in a 4-factor solution.

Finally, a multigroup analysis was carried out between groups reaching a full metric and scalar invariance. Figure 1 shows the final factor structure, in terms of factor loading for the whole data set. The goodness of fit, as can be seen in Table 6, is depicted by optimal values: χ^2^ = 546.260; *p* < 0.001; χ^2^/df = 3.35; IFI = 0.87; CFI = 0.90; RMSEA = 0.08.

**TABLE 6:** Goodness-of-fit on the analysis of invariance.

**Model**	**χ^2^**	**df**	**χ^2^/df**	**CFI**	**RMSEA**
Model 1: Configural invariance	770.133	326	2.362	0.862	0.067
Model 2: Full metric invariance	788.746	342	2.306	0.891	0.066
Model 3: Full metric and scalar	806.752	352	2.292	0.890	0.066
Model 4: Invariance on Residuals	864.563	373	2.318	0.881	0.066

## Discussion and Conclusion

The aim of this study was to analyze the psychometric properties of the Gratitude Questionnaire–20 item (G20) with a Prolific (ProA) user and English-speaking sample pool. The results indicate that the structure of the scale behaves in a similar way to that obtained in its construction in Spanish (Bernabé-Valero et al., 2014). The four components (IG, GS, RG, and EG) correlate significantly with each other and with the total scale, obtaining reliability indices even higher than those obtained with the Spanish sample.

These results are corroborated, after the multilevel CFA, grouping the participants in *low crowdsourcing use* and *high crowdsourcing use*, showing an optimal adjustment of the scale (full metric scalar) for the two groups. Although small differences have been found in the scores between the two groups in the subscales IG and EG, these differences show a very low effect size. Thus, we did expect groups did not affect the level of invariance.

The value of the Pearson coefficient obtained by correlating the G20 subscales with the GQ-5 scale suggests cross-cultural effects of gratitude, since no significant correlations have been obtained between both constructs. Our findings are congruent with recent studies about the cross-cultural differences in the personal understanding and expression of gratitude (Morgan et al., 2014; Mendonça et al., 2018; Mercon-Vargas et al., 2018).

Mendonça et al. (2018) studied the differences in the expression of gratitude in children and adolescents across seven societies (Brazil, China, Guatemala, Russia, South Korea, Turkey as representative of autonomous-related cultures, and the United States as representative of an autonomous-separate culture) considering different types of gratitude; verbal, concrete, and connective gratitude. They found that children in the US express higher levels of concrete gratitude, while in Asian cultures, as well as in the Eastern European countries, children are more prone to express connective gratitude. The authors also found that verbal gratitude is the predominant way in which Guatemalan children express gratitude. Mendonça et al. conclude that a cultural framework is necessary to avoid over-generalization when studying the development of gratitude as a virtue.

Another reason for the divergence found between the scales could be due to the fact that one-dimensional scales such as the GQ-5 sometimes do not work in cross-cultural studies since there are divergent cultural factors that cannot be considered in short scales. Morgan et al. (2014) study the differences between the United States and the United Kingdom and consider that gratitude may contain a common core with culturally ubiquitous features, in addition to socially constructed elements that change depending on the culture being studied (Morgan et al., 2014, p 25). They find that the three most used scales for assessing gratitude, the GQ-6 (McCullough et al., 2002), the GRAT (Watkins et al., 2003), and the Appreciation Scale (Adler and Fagley, 2005), focus mainly on the emotional component of gratitude and consider that this is not enough to be able to measure gratitude across cultures.

Following these findings, and in relation to the understanding of gratitude in each culture, we hypostatize that in our study when Spanish and American participants rate the degree to which they feel grateful (both in the GQ-5 and G20 items), it is possible that they understand certain elements differently. This circumstance would explain why there was a significant correlation between the G20 scale and the GQ-5 with a Spanish sample (Bernabé-Valero et al., 2014) but a significant correlation did not exist between the G20 subscales and the GQ-5 with an American sample.

In fact, although the GQ-5 is widely validated, the proposal made by Wood et al. (2008), in which the concept is broadened by considering it a higher order factor, suggests that gratitude could include more aspects than those measured by the GQ-5. That is why the need for a more comprehensive instrument was raised (Bernabé-Valero et al., 2014), one that would include the different basic and specific processes that lead to gratitude (e.g., the appreciation of gifts, the resignification of suffering, etc.) or its behavioral manifestation (e.g., the expression of gratitude, rituals of thanks-giving, etc.). Subsequently, the construction of the G20 contemplated the most relevant and current theoretical contributions on the construct of gratitude, trying to integrate various proposals. The scale expands and includes the construct’s cognitive, evaluative, emotional, and behavioral processes.

With a factor for transpersonal gratitude and two items (5 and 6) for interpersonal gratitude, the G20 reflects Seligman’s conclusion that expressing gratitude is necessary for the process of gratitude to be effectively concluded (Seligman, 2003). Thus, as Seligman proposes, the G-20 includes elements related to (1) becoming aware of gratifying experiences and never taking them for granted, and (2) taking the time to express gratitude.

In the G20, gratitude is also assessed in situations in which the experiences are not pleasant, but generate suffering, thus expanding the affective valence of the object of gratitude. This last contribution is crucial, since suffering is a reality in the lives of many people, but it does not necessarily diminish the ability to appreciate and be grateful for the positive aspects that, despite it, life continues to offer and that can be experienced as gifts.

Finally, in the G20 the different agents toward gratitude are made explicit. McCullough et al. (2002) include in their definition of gratitude that it is a response to the contribution by others to the well-being of the receiver, but the GQ-5 has been criticized for not imposing restrictions on its construction, since none of its items make explicit the contribution of an agent (Anderson, 2005). By contrast, the G20 makes explicit the different agents toward gratitude. In the G20, personal agents are specified in the IG subscale (e.g. “someone I hardly know helps me,” “someone does me an important favor,” “I get from people close to me”) and transpersonal agents in the RG subscale (e.g., “I thanked God or good fortune for it”).

The G20 with four factors has been able to be replicated using an American sample with excellent psychometric properties, thanks to its theoretical construction that allows it to expand the measurement by including elements not contemplated in other questionnaires. Future cross-cultural investigations may help to draw a more concise map of which elements differ, and which are common among different cultures.

Regarding gender, significant differences in gratitude have been found between men and women, which is consistent with previous studies (Baumgarten-Tramer, 1938; Levant and Kopecky, 1994; Krause, 2006; Kashdan et al., 2009; Bernabé-Valero et al., 2014; Roa-Meggo, 2017). Specifically, in the present study, women have obtained a significantly higher average in interpersonal aspects but not in the rest of the G20 components or in the total score of the scale. The reason may be that some men may understand the expression of gratitude in interpersonal relationships as evidence of vulnerability and weakness that can jeopardize their masculinity and social position. Consequently, they may adopt an avoidant attitude toward gratitude, inhibiting its expression (Kashdan et al., 2009). In contrast, women, who on average are more sensitive to interpersonal relationships, emotions, and behaviors whose goal is to create and maintain meaningful social relationships (Zacarés et al., 2009), perceive gratitude as more functional and as an advantage in their lives (Timmers et al., 1998; Schwartz and Rubel, 2005). In addition, women are more likely to acknowledge the goodwill of others, express appreciation, and reinforce the likelihood of these acts being repeated creating a lasting social resource (Kashdan et al., 2009).

The present work is based on a representative sample in terms of demographic variables (age, gender, educational level, employment, and marital status), overcoming the limitations of the standard student samples. The psychometric properties of the G20 with English speaking crowdsourcing users were similar to the properties obtained in the original Spanish form, with no specific biases being found in this type of sample.

In short, the results of this study allow us to conclude that the Gratitude Questionnaire–20 Items (G20) adapted to English with an American sample, is a psychometrically strong instrument to measure gratitude using crowdsourcing platforms for data collection and, therefore, a reference and useful tool in future research.

Digitization has ushered in a new era by multiplying the possibilities for much more efficient research. The English adaptation of the G20 opens a pathway to extend internationally its use for the measurement of gratitude and furthermore, for the advancement of cross-cultural studies.

## Data Availability Statement

The raw data supporting the conclusions of this article will be made available by the authors, without undue reservation.

## Ethics Statement

The studies involving human participants were reviewed and approved by the Universidad Católica de Valencia San Vicente Mártir committee (number UCV2017-2018-28). The patients/participants provided their written informed consent to participate in this study.

## Author Contributions

GB-V conceived of the presented idea. GB-V, JB-M, and MG-M developed the theory and performed the computations. All authors discussed the results and contributed to the final manuscript.

## Conflict of Interest

The authors declare that the research was conducted in the absence of any commercial or financial relationships that could be construed as a potential conflict of interest.
