# Supplementary material for: Gratitude Questionnaire–20 Items (G20): A Cross-Cultural, Psychometric and Crowdsourcing Analysis
Source: Front Psychol. 2020 Dec 21;11:626330. doi: 10.3389/fpsyg.2020.626330 (PMC7779484; doi:10.3389/fpsyg.2020.626330)
Supplement: Supplementary file 1 [file Data_Sheet_1.docx]

# **APPENDIX**

**Gratitude Questionnaire-20 Items (G20)**

Read each of these statements. Then, mark the answer that best describes to what extent you agree with them. Please try not to leave any statement unanswered (1 = strongly disagree; 7 = strongly agree).

1. I feel grateful when someone I hardly know helps me and/or is kind to me.

2. I feel great joy when someone does me an important favour.

3. When someone gives me a little present, with no expectation of gain, I feel very grateful.

4. I appreciate it when someone makes a sacrifice for me, even if it is only a small gesture.

5. I usually thank others when they have done something which has benefited me.

6. If someone helps me, as a sign of gratitude, I try to be responsible and hold up my end.

7. I highly value the friendship and love I get from people close to me.

8. Although I define some of my experiences as a negative, I can appreciate and acknowledge what they contribute to my life.

9. Even in situations of real suffering, I can find some value and I understand that there is some kind of meaning in it.

10. Even after times in my life when I only experienced suffering, I can feel gratitude for having had the strength to get through them.

11. I am aware that there is suffering in my life that happens so I can learn, and I am grateful for it.

12. When I'm going through bad times, I try to think of the good things that I have, and I manage to feel grateful.

13. I realize that there are many things that I should be grateful for.

14. Every day I am aware that the little things in life that happen to me are a gift.

15. I appreciate the many things I have in my life (personal qualities, relationships with others, etc.).

16. On comparing myself to others I see that there are people much less fortunate than me, and I am grateful for what I have.

17. When I ask God * for help and I receive it, I usually remember those favours and give thanks.

18. On any occasion when something bad could have happened to me (accident, loss, etc.) but did not, I thanked God or good fortune for it*.

19. I frequently offer thanksgiving and/or prayers for what I have received (before meals, prayers, etc.).

20. The best way to be grateful for life is to try and be happy.

* The term "God" can be replaced by "Luck", "Life", "Universe", "Higher Forces", or any other that has a personal meaning as an existential referent.

Interpersonal Gratitude (IG): Items 1–7

Gratitude in the face of Suffering (GS): Items 8–12

Recognition of Gifts (RG): Items 13–16

Expression of Gratitude (EG): Items 17–20
